# The remarkable history of pneumococcal vaccination: an ongoing challenge

**DOI:** 10.1186/s41479-022-00097-y

**Published:** 2022-09-25

**Authors:** Daniel M. Musher, Ronald Anderson, Charles Feldman

**Affiliations:** 1 Department of Medicine (Infectious Diseases), Baylor College of Medicine, Houston, TX 77030 USA; 2 Michael E. DeBakey VA Medical Center, TX 77030 Houston, USA; 3 Department of Immunology, Faculty of Health Sciences, University of Pretoria, Pretoria, 0001 South Africa; 4 Department of Internal Medicine, Faculty of Health Sciences, University of the Witwatersrand Medical School, 7 York Road, Parktown, Johannesburg, 2193 South Africa

**Keywords:** Advisory Committee on Immunization Practices, Heat-killed whole cell vaccines, Invasive pneumococcal disease, Pneumococcal polysaccharide conjugate vaccine, Pneumococcal polysaccharide vaccine, Pneumococcus, Recombinant protein vaccines, Serotypes, *Streptococcus pneumoniae*, Vaccination

## Abstract

Although it varies with age and geographical distribution, the global burden of infection with *Streptococcus pneumoniae* (pneumococcus) remains considerable. The elderly, and younger adults with comorbid conditions, are at particularly high risk of pneumococcal infection, and this risk will increase as the population ages. Vaccination should be the backbone of our current strategies to deal with this infection.

**Main body: **This manuscript reviews the history of the development of pneumococcal vaccines, and the impact of different vaccines and vaccination strategies over the past 111 years. It documents the early years of vaccine development in the gold mines of South Africa, when vaccination with killed pneumococci was shown to be effective, even before the recognition that different pneumococci were antigenically distinct. The development of type-specific vaccines, still with whole killed pneumococci, showed a high degree of efficacy. The identification of the importance of the pneumococcal capsule heralded the era of vaccination with capsular polysaccharides, although with the advent of penicillin, interest in pneumococcal vaccine development waned. The efforts of Austrian and his colleagues, who documented that despite penicillin therapy, patients still died from pneumococcal infection in the first 96 h, ultimately led to the licensing first of a 14-valent pneumococcal polysaccharide in 1977 followed by the 23-valent pneumococcal polysaccharide in 1983. The principal problem with these*,* as with other polysaccharide vaccines, was that that they failed to immunize infants and toddlers, who were at highest risk for pneumococcal disease. This was overcome by chemical linking or conjugation of the polysaccharide molecules to an immunogenic carrier protein. Thus began the era of pneumococcal conjugate vaccine (PCV), starting with PCV7, progressing to PCV10 and PCV13, and, most recently, PCV15 and PCV20. However, these vaccines remain serotype specific, posing the challenge of new serotypes replacing vaccine types. Current research addresses serotype-independent vaccines which, so far, has been a challenging and elusive endeavor.

**Conclusion: **While there has been enormous progress in the development of pneumococcal vaccines during the past century, attempts to develop a vaccine that will retain its efficacy for most pneumococcal serotypes are ongoing.

## Introduction

The global burden of community-acquired pneumonia (CAP), in general, and infections with *Streptococcus pneumoniae* (the pneumococcus) in particular, have remained considerable [1, 2], albeit of unequal geographical distribution [3, 4]. Epidemiologic studies have emphasized that the impact of pneumococcal infections is substantial in older adults, with and without comorbid disease and in younger adults with comorbid conditions, and indicated that as the world’s population continues to age, these infections are likely to become even more problematic. Even with appropriate treatment, pneumococcal pneumonia has both acute [5] and long-term [6, 7] morbid effects. Thus, prevention should remain the backbone of our efforts. The aim of this manuscript is to review the history of pneumococcal vaccination and the impact of different pneumococcal vaccines and vaccination strategies that have been utilized in adults over the past 111 years.

### The early years of pneumococcal vaccine development

More than one hundred years ago, the South African gold mining industry asked British physician, Almroth Wright, who had previously worked on a vaccine against typhoid fever, to study vaccination against pneumococcal pneumonia in men working in gold mines located on the Witwatersrand [8]. Whether practical or altruistic, the motivation of the mine owners was simple. Potential miners seeking employment came from all around Africa and were housed in barracks. During the first four months of arrival, the monthly attack rate of pneumonia – nearly all of which was proven to be due to pneumococcus – was between 1 and 2%, and in men who were proven to be infected, the death rate ranged between 25 and 56%. Between 5 and 10% of men who began working in the mines died each year [8]. The observations of Wright and his colleagues [8] showed a substantial reduction in cases of pneumonia and in deaths following inoculation of varying doses of killed pneumococci [9].

### Initial experiments with pneumococcus as a vaccine

What was the basis for recommending inoculation with killed pneumococci? Remarkably, two decades earlier, and in a single year (1891), three different groups of investigators reported observations on induction of immunity by repeated inoculation of experimental animals with pneumococci [10]. Most influential was the work of Georg and Felix Klemperer [11], an uncle and nephew who showed that repeated inoculation of rabbits with killed pneumococci rendered them immune to subsequent challenge by that organism. Almost as if added as an afterthought, their article states that when they transferred serum, the humoral substance, from immunized to unimmunized rabbits, these animals were also protected against pneumococcal challenge. These seminal observations documented: (i) the induction of immunity by repeated inoculation with killed organisms; and (ii) the transferability of immunity with serum—the basic principle of humoral immunity.

The possible existence within organisms identified as pneumococci, of antigenically distinct groups, was suggested by Besancon and Griffon [12], and in 1910, Neufeld and Handel (cited in White [13], p.361) reported that type 1 and type 2 pneumococci could be distinguished by serologic reactions. In a meticulous study of all patients with lobar pneumonia treated at the Rockefeller Institute during three years, the great majority of whom were infected with pneumococcus, Dochez and Gillespie, in 1913 [14], used these serologic techniques to stratify the pneumococci they isolated into three groups (later called serotypes or types), numbered 1, 2 and 3 (78% of patients); they classified the remaining 22% of pneumococci as group (type) 4. Based on protection against pneumococcal challenge in mice, they found that types 1, 2 and 3 were serologically distinct; protection against pneumococcal challenge in mice was specific to each type and did not extend to challenge with other types.

### Initial vaccine studies in humans

Unfortunately, the absolute importance of type specificity was apparently not considered by the first investigators who tried to apply immunization in human subjects. The massive work of Wright et al., included a total of at least 60,802 miners. In most experiments, subjects were randomized 1:1 either to receive or not to receive vaccine; in some, the randomization was 2:1 [9, 15]. Overall, these investigators correctly interpreted their data as showing a nearly 50% reduction both in cases and in deaths due to pneumonia, results that we find impressive. Criticisms about methods of randomization are trivial when compared to the essential flaw in this enormous body of work, namely, that the investigators failed to consider which type(s) of pneumococci were used in the vaccine and which were prevalent as infecting organisms at the time of the vaccination program. Wright et al. directed most of their attention to different doses of killed organisms and tried to explain inconsistent results in some experiments on dosage, but there is simply no mention of pneumococcal types, which is understandable considering that Neufeld and Handler had only reported their results in 1910 [9, 15]. Attempts at vaccination by Maynard [16], also in South African miners, very likely yielded inconsistent results because of serotype mismatch between inoculated and infecting types. In 1913, Dochez and Gillespie [14] commented on these studies, stating that “heterogeneous collections of organisms are used, and difference in origin is the only standard of differentiation of the various strains employed”.

### Type-specific protection by pneumococcal vaccine

Working only a few years later, Lister, a protégé of Wright, supported the criticism by Dochez and Gillespie when he showed, in a study of 10,866 newly arrived miners, that type-specific vaccine was 100% effective in preventing infection due to pneumococcus types 1 and 2 [17]. When the camps were closed and then reopened, a new major outbreak was abruptly terminated with the use of a trivalent whole-pneumococcal vaccine.

Working in the United States, in 1917, Cecil and Austin [18] identified cases of pneumonia at military Camp Upton due to types 1, 2 or 3 pneumococcus. They inoculated varying doses of killed pneumococci from these types into a small number of subjects and studied agglutination of pneumococci and protection against mouse challenge by serum of vaccinees, as well as adverse effects of the vaccine. Based on these results, they chose to administer four weekly injections of 1–3 × 10^9^ killed pneumococci to 12,519 subjects. An additional 19,481 subjects, who were unvaccinated were also studied. About 14,610 of the controls matched the vaccinated subjects in having been present at the camp (although the durations of their stay were unstated). The remaining control subjects were newly arrived recruits. The authors did not state how they selected men who were to be vaccinated, but it seems fair to assume that no particular selection bias might have affected the outcome. Strikingly, no cases of type 1, 2 or 3 pneumococcal infections occurred amongst the 12,519 vaccinated subjects (ignoring a single case in whom infection developed the day after the first vaccination) compared to 18 of 14,610 of the ‘matched’ unvaccinated group, again showing the efficacy of type-specific pneumococcal vaccine. It is important to note that some of the efficacy shown in studies using whole, killed pneumococci may have been due to cell constituents other than capsular, although the early failure to demonstrate a beneficial effect argues against that possibility.

### Vaccination with purified capsular polysaccharides

In a remarkably logical series of experiments over the next two decades, pneumococcal capsule was shown to be the immunizing substance. In 1917, Dochez and Avery reported a “soluble specific substance” in urine and serum of infected patients [19] and in broth cultures of pneumococcus [20]; this substance precipitated when exposed to serotype-specific antiserum. Heidelberger and Avery tentatively suggested that this substance was the capsular polysaccharide (CPS) [21] and appeared to confirm that suggestion by showing that different types of pneumococcus had capsules with chemically different polysaccharides [22]. In an elegant series of experiments, Felton [23] showed that: (i) CPS was responsible for serotypes; (ii) antibody to this polysaccharide was responsible for the agglutination reaction of pneumococci; and (iii) immunization with CPS induced immunity to pneumococcal infection.

Large clinical trials of pneumococcal vaccine followed. In the winter of 1933–4, Ekwurzel, Felton et al. [24], vaccinated 3,126 members of the Civilian Conservation Corps with a preparation of equal parts of CPS from type 1 and type 2 pneumococcus. No pneumonia occurred, compared to eight cases among approximately 9,000 unvaccinated individuals (Table 1). Over the next three winters, 39,621 additional Corps members were vaccinated and 44,494 were not. Each year, the investigators continued to carefully observe their subjects, but recruitment and follow-up times were not uniform, and all of these studies were carried out over relatively short time periods. In the winter of 1937–8, Smillie and colleagues [25] used Felton’s recommended dose of type 1 pneumococcal polysaccharide to abort an outbreak of type 1 pneumococcal pneumonia at a state hospital in Worcester. Shortly thereafter, they repeated this maneuver at an annex to the main hospital. They properly stated that, “We cannot affirm, of course, that the antigen stopped the outbreak… [it] might have stopped simultaneously”.

**Table 1:** Vaccination of members of the civilian conservation corps, 1934–1937

	**Vaccinated**	**Non-vaccinated**	**Comments**
1933–4	3,126 (0)	9,000 (8)	No pneumonia in vaccinated versus 8 cases among unvaccinated
1934–5	14,000 (13)	12,000 (23)	Vaccine appeared to be protective, but results difficult to interpret because of heterogeneity amongst the groups
1934–5	14,881 (18)	18,000 (39)	No type 1 or type 2 pneumonia in the vaccinated group versus 6 of each type among the controls
1936–7	10,740 (13)	14,494 (24)	Much better follow-up of subjects No pneumonia due to type 1 or 2 pneumococcus in the vaccinated group versus 13 cases in the controls

Vaccination with type 1 and type 2 capsular polysaccharides. Results are shown as numbers in each group with patients who developed pneumonia during the period of observation shown in parentheses

From reference [24]

During the Second World War, outbreaks of pneumococcal pneumonia in US army camps created an opportunity to do well-controlled studies of pneumococcal vaccine. In 1944, MacLeod et al. [26], used a vaccine that contained polysaccharides from pneumococcus types 1, 2, 5 and 7 in a clinical trial involving 17,035 soldiers at a US Army Technical School. With self-randomization, 8,586 men received vaccine, and 8,449 served as controls. They were followed for an average of about 6 months, and the occurrence of all cases of pneumococcal pneumonia was documented (Table 2). The incidence of pneumonia due to types 2 and 7 was significantly reduced (statistical analysis is added; this was not reported in the original paper); pneumonia caused by nonvaccine types was unchanged.

**Table 2:** Vaccination of soldiers at a US army technical school, 1942–4

		**Number of pneumonia cases**	
**Serotypes included in the vaccine**		**Controls** **(*n* = 8449)**	**Vaccine recipients** **(*n* = 8586)**
1	yes	2	2
2	yes	14	1*
4	no	6	8
5	yes	4	1
7	yes	6	0*
12	no	25	21
other	no	28	27

From reference [26]

^*^p ≤ 0.05

### Pneumococcal vaccination in the antibiotic era

With the discovery of penicillin, interest in vaccines to prevent pneumonia waned; a systematic review of the etiology of pneumonia disclosed only four publications on the subject between 1946 and 1970 [27]. The assumption was that the problem would largely be eliminated by use of this antibiotic. Austrian and Gold [5], however, showed that, despite treatment with penicillin, deaths from pneumococcal pneumonia were unchanged in the first 96 h of therapy. In other words, penicillin reduced, but, by no means eliminated deaths due to pneumococcal pneumonia, and Austrian made it his life’s work to reactivate interest in pneumococcal vaccination [28]. He and his colleagues developed two pneumococcal polysaccharide capsular vaccines (PPSV) preparations, one containing six and one containing 12 capsular types [29], which Smit et al. [30] studied in two separate randomized controlled studies in South African miners. The vaccine efficacy for types contained in the vaccine averaged 84%. In response to this work, in 1977, Merck licensed a vaccine containing capsular polysaccharides from 14 pneumococcal types and followed this in 1983 with one containing 23 capsular polysaccharides (PPSV23 [Pneumovax®]), which is still marketed today.

In the two ensuing decades, many investigations of the efficacy of pneumococcal vaccine were carried out – including randomized control trials (RCTs), observational, case control, and indirect cohort studies. These were summarized in two widely cited meta-analyses published in 2008 by Moberley et al. (a Cochrane Review [31]), and in 2009 by Huss et al. [32]. Moberley and her colleagues included in their final analysis 22 RCTs involving 48,566 subjects and 7 non-RCTs involving 62,294 subjects. These authors concluded that PPSV reduced the risk for bacteremic pneumococcal pneumonia, non-bacteremic pneumococcal pneumonia, and all invasive pneumococcal disease (IPD) by 87%, 73% and 82%, respectively. Huss [32], a statistician, excluded all but five studies of PPSV for statistical reasons, and concluded that this vaccine produced no significant benefit; this analysis gave heavy weight to two studies that met all statistical criteria for design, but used non-validated methods for diagnosing pneumococcal infection. A second Cochrane Review by Moberley et al., in 2013 [33], reached conclusions similar to those in their report of 2008. It is worthwhile however, to give examples of studies that showed no benefit from PPSV23. In a carefully designed and executed RCT, Simberkoff et al. [34] found that, in an older male population with many comorbid conditions, there was no apparent protection from PPSV23, but this study was underpowered.

Using a case–control design, Shapiro et al. [35], showed that protection of young adults by PPSV23 at 3 years exceeded 90% and remained at > 85% for 5 years of observation. However, with each decade of increase in age of the subjects, protection declined, such that in the oldest group, no protection was observed five years post-vaccination.

Recent studies of PPSV23 have often shown much less protection than did earlier ones (Table 3) [36–41]. Possible reasons for reduced vaccine efficacy of PPSV23 in recent years include: (i) persistence of types for which vaccine (PPSV or protein-conjugate vaccine [see below]) does not appear to stimulate good protection, e.g., serotypes 3, 19A; (ii) decreasing prominence of PCV13 strains from the population with opening of an ecologic niche for other pneumococcal types; and (iii) the consequent emergence of strains not covered by PPSV23, e.g., types 15A, 23B, 35 and 38 [34].

**Table 3:** Recent studies of vaccine efficacy of PPSV23

	**Vaccine efficacy in pneumococcal pneumonia (PP)**^**a**^		
**Author, year**	**All PP**	**VT PP**	**Comment**
Suzuki 2017 [36]	27%	34%	CC, ≥ 65 years
Djennad 2019 [37]	27%		CC, ≥ 65 years
Kim 2019 [38]		21%	CC, 65–75 years
Lawrence 2020 [39]		24%	CC, persons ≥ 16 years
Vila-Corcoles 2020 [40]	0%		Pop-based cohort ≥ 50 years
Masuda 2021 [41]	61%		CC, ≥ 65 years, CRD

*PP* Pneumococcal pneumonia, *VT* Vaccine-type, *CC* Case control study, *Pop* Population, *CRD* Chronic respiratory disease

^**a**^ Most of these studies show that protection decreases as age, and time since vaccination, increase

### Protein conjugate vaccines

The principal problem with PPSV, as had been the case with the polyribosyl ribitol phosphate (PRP) capsular vaccine for *Haemophilus influenzae* type b*,* was that it failed to immunize infants and toddlers, the subjects who were also at highest risk for pneumococcal disease. To overcome this problem, Schneerson et al. [42], in 1980, showed that chemical linking or conjugation of PRP to a protein (they used CRM_197_, a non-toxic recombinant variant of diphtheria toxin) rendered it immunogenic. Within a few years, widespread vaccination of infants with CRM_197_-conjugated PRP almost eliminated *H. influenzae* type b meningitis from the population. Inspired by this success investigators at Wyeth, conjugated CPS of the seven most common types of *Streptococcus pneumoniae* to CRM_197_. In a seminal study [43], 37,868 infants in the Kaiser Permanente health care system were randomized to receive the 7-valent conjugate pneumococcal vaccine (Prevnar7®) or meningococcal polysaccharide (as a placebo). The incidence of pneumococcal meningitis was reduced by > 95% in infants who were fully vaccinated, and the type-specific vaccine efficacy against all pneumococcal invasive disease exceeded 97%. The lesser effect on all-cause pneumonia or otitis media reflects the multiplicity of etiologic organisms in these infections.

**Fig. 1:**
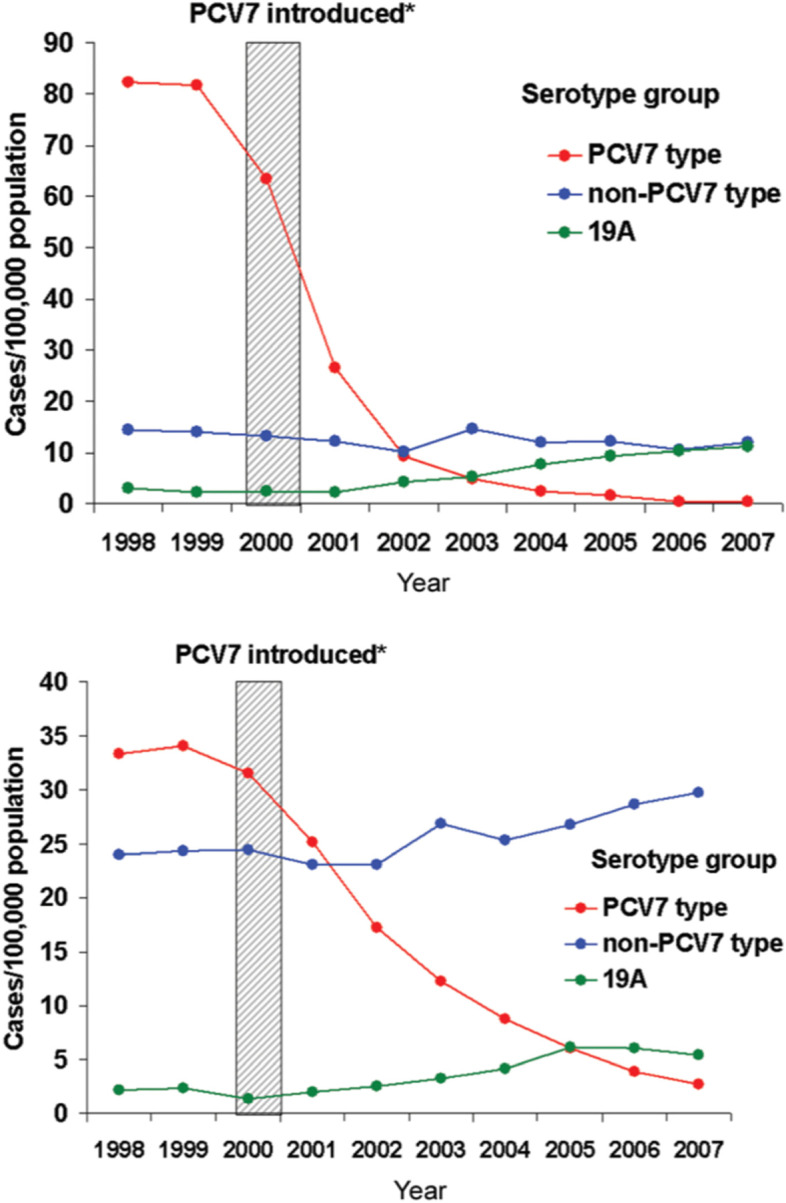
Effect of PCV7 on IPD in children < 5 years (direct effect; upper panel) and adults > 65 years (herd effect; lower panel). Note increase in non-PCV7 types, especially 19A. * From reference [46], by permission of Oxford University Press

As had been suggested by some observers [44, 45] the establishment of mucosal immunity by PCV greatly reduced pneumococcal carriage and, therefore, pneumococcal transmission in vaccine recipients. This led to a striking secondary effect, namely, reducing or nearly eliminating infection due to vaccine strains in the population at large (Fig. 1) [46]. A similar result would later follow widespread use of a 13-valent conjugate vaccine [47].

Between 2008 and 2010, in a large and well-designed clinical trial, Bonten et al. [48], randomized 84,496 Dutch adults ≥ 65 years of age to receive a conjugate vaccine that contained CPS from 13 pneumococcal serotypes conjugated to CRM_197_ (PCV13) or placebo. This study excluded adults with immunocompromising conditions and subjects who were placed on an immunosuppressive drug from the final analysis. PCV13 reduced IPD and non-bacteremic pneumococcal pneumonia due to vaccine-type pneumococci (diagnosed using a serotype-specific urine antigen detection test) by 75% and 45%, respectively [48]. At the time of this trial, < 2% of Dutch adults had received a pneumococcal vaccine, childhood vaccination with PCV7 was in its early stages, and PCV13 was not yet approved for use in children, rendering comparison with the situation in the US problematic. The trial did not compare PCV13 with PPSV23, so any conclusion about the relative benefits remains inferential. While some patients in this study did develop immunosuppressing conditions, the study was not designed to show efficacy in such patients, and the number of these patients was too low to draw any meaningful conclusions regarding PCV13 efficacy in this population.

An additional population-based study of effectiveness of PCV13 vaccination in preventing hospitalization for vaccine-type CAP in adults > 65 years was undertaken in Louisville, Kentucky during 2015–2016 [49], at which time a substantial proportion of infants and children had received PCV13. Cases were those hospitalized with CAP due to PCV13 serotypes (based on culture or the serotype-specific urinary antigen detection test), whereas controls were those hospitalized with CAP, without infections caused by any of the PCV13 serotypes. This study included adults with or without compromised immunity and cases we would previously have labeled as healthcare-associated pneumonia. Vaccine effectiveness was 73% for all PCV13-type CAP and 68% for non-bacteremic PCV13-type CAP. To our knowledge, no clinical trial has directly compared vaccine efficacy of PCV13 to that of PPSV23.

Immunogenicity and safety comparisons between these two vaccines, including at least one systematic review and meta-analysis, have indicated that PCV13 elicits a better immune response among adults compared with PPSV23, while having a similar safety profile [50]; however, these studies all presented data at one month after vaccination. Supplementary data in Jackson et al. [51], however, showed that at 12-months post vaccination, there was no discernible difference in opsonophagocytic effect of serum from recipients of PCV13 or PPSV23.

There have also been cost-effectiveness studies of PCV13 versus PPSV23. Earlier studies, before the full impact of PCV13 on non-bacteremic pneumococcal pneumonia, or the magnitude of PCV13-associated indirect herd effects were known, indicated, respectively, that adding PCV13 to adult pneumococcal vaccination was favored compared with PPSV23 [52], and that cost-effectiveness of PCV13 in adults aged 50 years and older was comparable to other vaccine interventions [53]. Other studies, including one in a population in which a significant number of adults had pre-existing HIV infection, showed PCV13 to be cost-effective compared with PPSV23, both in the population as a whole and in particular in the HIV-infected population [54]. It seems highly unlikely that PCV13 followed by PPSV23 will be cost effective in countries where PCV13 has been used widely in infants and young children, since these strains have largely disappeared from the adult population.

### The advisory committee for immunization practices (ACIP) recommendations for pneumococcal vaccinations in adults

The milestones in the ACIP recommendations for pneumococcal vaccination in adults are shown in Table 4, starting with updated recommendations for use of PPSV23 in adults in 2010, followed thereafter by recommendations initially for use of PCV13 in adults aged 50 years and older (this recommendation has now been archived) and then later recommendations for use of PCV13 followed by PPSV23, based on risk factors and then on age [55–60].

**Table 4:** Milestones in the ACIP recommendations for use of pneumococcal vaccines in adults

2010:	ACIP updates recommendations for PPSV23 in adults – 5 years apart; max 2–3 doses in a lifetime
2011:	FDA approved PCV13 for adults ≥ 50 years – given always 1 year after latest PPSV23 dose^a^
2012:	ACIP recommendation published for the use of PCV13 followed by PPSV23 ≥ 8 weeks later in adults ≥ 19 years with immunocompromising conditions, functional or anatomic asplenia, cerebrospinal fluid leak, and cochlear implant (20 June 2012)^b^
2014:	ACIP recommendation published for use of PCV13 in adults ≥ 65 years followed by PPSV23 6–12 months later (13 August 2014)^b^
2015:	ACIP changes time interval between PCV13 and PPSV23 in immunocompetent adults ≥ 65 years to one year (4 September 2015)^b^
2019:	ACIP reconsiders use of PCV13 in adults ≥ 65 years, recommending a single dose of PPSV23 and not routine use of PCV13 in these adults who do not have an immunocompromising conditions, cerebrospinal fluid leak, or cochlear implant. If a decision to administer PCV13 is made, PCV13 should be administered first, followed by PPSV23 at least 1 year later. If there are immunocompromising conditions, functional or anatomic asplenia, cerebrospinal fluid leak, or cochlear implant then PCV13 should be given first followed by PPSV23 ≥ 8 weeks later^b^

*sACIP* Advisory Committee on Immunization Practices, *PPSV23* 23-valent pneumococcal polysaccharide vaccine, *PCV13* 13-valent pneumococcal conjugate vaccine

^a^ This recommendation has now been archived

^b^ These recommended schedules described are mainly for vaccine naïve individuals; if these individuals have been vaccinated previously with either of the vaccines, there are different schedules, as indicated in the references

From references [55–60]

In 2015, the ACIP recommended PCV13 followed a year later by PPSV23 for all adults ≥ 65 years of age. The recommendation was based on several assumptions that, as Musher [61] pointed out elsewhere, may not have been well-based in fact; (i) PCV13 stimulates higher levels of anti-CPS antibody than PPSV23; (ii) this antibody persists for longer intervals; (iii) PCV13 primes the immune system for a booster response by PPSV23; and (iv) PCV13 more effectively protects immunocompromised and elderly adults than does PPSV23. Perhaps the most important reason not to follow such a recommendation in the US was the consideration that once a conjugate polysaccharide vaccine was routinely used in children, those strains would disappear from the population; but this has not been consistent in many countries outside the US, as discussed further below.

In 2019, the ACIP down-graded the recommendation, indicating that in the elderly without CSF leak, cochlear implant or immunocompromising conditions, only PPSV23 should be given routinely, leaving the matter of whether PCV13 should be given in addition, to shared decision-making by patients and doctors. However, in the case of the elderly with CSF leak, cochlear implant or immunocompromising conditions, the recommendation for the use of PCV13 followed by PPSV23 persists [60]. The decision in 2019 was based on a review of the circumstances that existed in the US regarding residual PCV13-type pneumococcal pneumonia in adults aged ≥ 65 years of age. The use of PCV13 in children had resulted in sharp declines in, and historically low levels of PCV13-type pneumonia in the elderly, with little added benefit from additional direct vaccination. However, it was recognized by the ACIP authors that in certain circumstances the elderly may be at potentially greater risk of exposure to PCV13 serotypes. These would include those living in nursing homes or long-term care facilities, or in settings with low PCV13 uptake, or those intending to travel to an area with no PCV13 immunization program, all of whom may benefit additionally from direct vaccination [60].

Some additional factors need to be considered regarding these recommendations (Table 5) [62–75]. Environmental factors may affect the burden of pneumococcal disease and the immune response to vaccines [62]. Differences in surveillance systems, local epidemiology and PCV programs may hamper comparisons among different regions [63]. Furthermore, risk factors for IPD vary in frequency in different parts of the world, and the different risk factors have different degrees of risk for IPD [64]. For example, studies from the US [65] and South Africa [66] have indicated that the incidence and risk factors for IPD and CAP are higher in people living with HIV compared with the general population, even when antiretroviral therapy has suppressed the virus and the CD4 cell count has normalized. These findings strengthen the case for continuing intensive vaccination programs in this population. A cost-effectiveness study comparing PCV13 and PPSV23 vaccines in South African adults, indicated that both in the public and private sectors, adult vaccination with PCV13 was cost-effective compared with PPSV23, in the whole population and especially those persons living with HIV infection [54]. In this context, some studies have confirmed immunological evidence for benefit of pneumococcal vaccination with PCV13 followed by PPSV23 versus PPSV23 alone in HIV-infected adults [67], but observations were confined to 8 weeks post-vaccination.

**Table 5:** Additional aspects that need consideration in the elderly

• Burden of pneumococcal disease varies in different regions
- Different rates of risk factors such as smoking, HIV, other comorbidities in the population or community
• Herd protection may be different geographically as may the residual burden of disease
- Different susceptibilities of the hosts, which childhood vaccines are used, and their coverage rate, force of transmission, proportion of disease caused by vaccine serotype
• Does herd protection have a limit?
• Decline of PCV13 serotypes in adults is attenuated by older age and comorbidity
• Underestimation of residual pneumococcal disease
- Especially of non-invasive infection, and/or when using conventional microbiological methods only
• Serotype replacement disease
- Varies in different countries

From references [62–75]

There has also been a suggestion that in some European countries, such as Spain, Germany and Italy, as well as South Africa, as opposed to the US, a limit to herd protection (“herd limit”) may have occurred prior to the COVID-19 pandemic, with serotypes causing pneumococcal infections in older children and adults [68–73]. Furthermore, with regard to pneumococcal disease, Pelton and colleagues have shown that the decline in pneumococcal disease even in older US adults following childhood vaccination, is attenuated by increased age and the presence of comorbidities [74]. In addition, Pelton and others also documented in German children and adults, that the risk for all-cause pneumonia (used as a proxy for pneumococcal pneumonia) was greater in the presence of comorbidities, perhaps even exceeding that in some of the recognized high-risk conditions [75].

The recent licensing of two new pneumococcal conjugate vaccines (PCV15 and PCV20) for use in Europe and the USA has resulted in an update of ACIP recommendations in 2022 [76]. In addition to the PCV13 serotypes, PCV15 vaccine also contains serotypes 22F and 33F, while PCV20 contains additional serotypes 8, 10A, 11A, 12F, 15B, 22F, and 33F. The recommendations for use of these newer conjugate vaccines were based on appropriate immunogenicity and safety studies with each vaccine, as well as cost-effectiveness studies. The current recommendations have resulted in significant simplification of pneumococcal vaccination recommendations in adults, who are vaccine naïve, but there still remain questions regarding the best options for subsequent vaccination with these newer vaccines in adults who have previously received either PPSV23 and/or PCV13.

For vaccine-naïve individuals, the new recommendations [76] are that adults 19–64 years of age with any underlying medical condition (comorbidities, or high-risk conditions or immunocompromise), and all persons 65 years of age and older (including those with or without any of the medical conditions indicated above), should receive one dose of PCV20 alone, or one dose of PCV15 followed by PPSV23 one year later. In patients with immunocompromising conditions or CSF leak or cochlear implant, the interval between PCV15 and PPSV23 could be shortened to 8 weeks. For those that have received only PPSV23 previously, either PCV15 or PCV20 can be given one year later, with a second dose of PPSV23 not being required following PCV15 in these patients. It is not clear what the incremental public benefit would be for providing PCV15 or PCV20 in those that have previously had PCV13 or PCV13 and PPSV23 and the current suggestion is to complete the previously recommended PPSV23 series. The recommendations for PCV15 and PCV20 will probably need to be reevaluated in light of prevailing availability of vaccines, the frequency with which they have been used in the pediatric population, and prevalent infecting strains.

Even beyond these new recommendations, a number of questions remain, and there are even newer conjugate vaccines in development. One of the ongoing debates is whether the best way to protect adults and the elderly is to vaccinate children with these new conjugate vaccines and rely on herd protection for adults or, alternatively, whether these newer vaccines should be used primarily in adults [77–80]. There are two issues that need consideration regarding this, one being residual vaccine-type disease, and the other, possibly the greater problem, being the emergence of non-vaccine serotypes [77, 78]. For residual vaccine-serotype disease, introduction of the new conjugate vaccines directly into the adult program may adequately deal with this. However, because children are the main reservoir for pneumococci, introducing these new conjugate vaccines into childhood vaccine programs may not only reduce or eliminate carriage with vaccine-serotypes in children, but could also lead to an increase in colonization of children with non-vaccine serotypes, posing a risk of subsequent disease with these non-vaccine serotypes in both adults and children [77]. While serotype replacement disease does not appear to have been much of a problem in the US, it has been in Europe and other areas. Clearly, to address these issues, development of vaccines that are effective against all pneumococcal serotypes, as well as non-encapsulated pneumococci, would be ideal [79].

### Recombinant protein-based pneumococcal vaccines

Serotype replacement following administration of current PCVs has not only driven the development of next generation, extended PCVs, which contain CPS derived from emerging, opportunistic serotypes of the pathogen [81, 82], but also the formulation of novel vaccines based on recombinant pneumococcal proteins [83–87]. This latter strategy hinges on the immunogenicity of highly conserved, broadly serotype-independent pneumococcal virulence factors, particularly surface adhesins. Notwithstanding a possible lesser dependence on the limited serotype coverage provided by expensive, multivalent CPS-based vaccines, recombinant protein vaccines have the additional potential advantage of conferring protection against pneumococci of any capsular type or even against non-encapsulated strains. Various types of pneumococcal protein-based experimental vaccines have been formulated, including those that are comprised of: (i) recombinant proteins per se, either individually or in combination; (ii) pneumococcal fusion/hybrid proteins; (iii) nanoparticle-packaged fusion/hybrid proteins; (iv) recombinant protein-boosted PCVs; and (v) experimental PCVs in which capsular polysaccharides are linked to pneumococcal carrier proteins, as opposed to diphtheria or tetanus toxoids. Because these concepts and vaccines are new and in development, the work that we will cite in this section will have been largely carried out in animal and/or early phase clinical studies.

### Exclusively protein-based vaccines

Pneumococcal proteins that have attracted considerable attention as potential vaccine candidates include: (i) pneumococcal choline-binding protein A (PcpA); (ii) a second, distinct choline-binding protein, known as choline-binding protein A (CbpA); (iii) pneumococcal surface protein A (PspA); (iv) pneumococcal surface adhesin A (PsaA); (v) histidine triad protein D (PhtD); and (vi) the pneumococcal pore-forming toxin released on autolysis of the pathogen, pneumolysin, formulated as the detoxified pneumolysoid of which there are several variants known, for example, as Δ146Ply, dPLY or PlyD1. The activities of these pneumococcal virulence factors are summarized in Table 6. Although invariably protective in preclinical murine models of experimental infection, vaccine formulations of these recombinant proteins either individually, or in combination (for example PcpA + PhtD or PcpA + PhtD + dPly) [83, 88], have failed to provide adequate protection against pneumococcal disease in humans, presumably because of the efficacy of the pneumococcal capsule in preventing access of host immune defense mechanisms to the surface and internal protein antigens of the pathogen that are targeted by the vaccines as demonstrated in an infant mouse model of pneumococcal colonization [89].

**Table 6:** Prominent pneumococcal virulence proteins with vaccine potential

Pneumococcal Protein	Virulence activities
Choline-binding protein A (CbpA)	Adhesin that binds to the polymeric immunoglobulin receptor, the laminin receptor and complement component 3
Histidine triad protein D (PhtD)	A factor H (FH)-binding protein that interferes with activation of the alternate complement pathway
Pneumococcal choline-binding protein A (PcpA)	Adhesin that also suppresses innate immune mechanisms
Pneumococcal surface adhesin A (PsaA)	Adhesin that also functions as a manganese permease complex
Pneumococcal surface protein A (PspA)	Adhesin that also inhibits recognition of the pneumococcus by C-reactive protein; also inactivates both factor H (FH) of the alternative complement pathway and lactoferrin
Pneumolysin	The membrane pore-forming eukaryotic cell cytotoxin of the pneumococcus

### Fusion/hybrid protein vaccines

More recently, artificially engineered pneumococcal recombinant fusion proteins and hybrid protein/peptide construct vaccines that merge several protein antigens into an individual protein with improved immunogenicity have been described. These include: (i) the Δ146Ply-SP0148 fusion protein [SP0148 is a component of the ABC (ATP transport cassette) transporter] [90]; (ii) another single fusion protein construct, YPT-L460D-NEEK (YLN), that links the polymeric immunoglobulin receptor- and laminin receptor-binding domains (YPT and NEEK, respectively) of CbpA, with the pneumolysoid, L460D [85]; and (iii) the cCHP nanogel-trivalent PsaA vaccine, a nasally administered vaccine, comprised of three PspA fusion constructs derived from three different pneumococcal strains [84, 91, 92]. Each fusion protein consists of the N-terminal α-helical coiled-coil domain (αHD) of PspA from each strain of the pathogen, fused with the central proline-rich domains (PRDs) of the other strains. These proteins are packaged in an inert, cationic nanogel known as cCHP (cationic cholesteryl pullulan nanogel), which adheres to mucosal surfaces, enabling effective delivery of vaccine antigens, potentially augmenting mucosal immunity and restricting nasopharyngeal colonization by the pneumococcus.

All three of these novel vaccines have demonstrated protective efficacy in preclinical animal models of experimental pneumococcal infection. However, clinical efficacy remains to be established.

### Augmentation of PCVs via the addition of recombinant pneumococcal proteins

Boosting the immunogenicity and serotype coverage of PCVs via the addition of recombinant pneumococcal proteins is also a promising strategy to improve vaccine efficacy. Formulation of one such experimental vaccine involved addition of recombinant PhtD and dPly to a PCV10 known as Synflorix™ (GSK, Belgium), comprised of CPS derived from pneumococcal serotypes 1, 4, 5, 6B, 7F, 9 V, 14, 18C, 19F and 23F conjugated to protein D of non-typeable *Haemophilus influenzae* [93]. Administration of this vaccine to healthy Gambian infants was, however, no more effective than the pneumococcal, protein-free, comparator vaccine in reducing the prevalence of nasopharyngeal carriage of non-vaccine serotypes of the pathogen. Similar findings were observed in the setting of prevention of acute otitis media in healthy Native American infants immunized with PCV13 boosted with PhtD/dPly, which was no more effective than PCV13 alone [94]. 

### Formulation of PCVs with recombinant pneumococcal carrier proteins

The development of PCVs in which CPS are linked to recombinant pneumococcal carrier proteins, as opposed to diphtheria/tetanus toxoids, represents an alternative, albeit challenging, strategy to augment the immunogenicity and serotype coverage of PCVs. In this context, several recent studies are noteworthy.

In the first of these [95], Reglinski et al., described a novel, seemingly inexpensive, procedure to produce novel PCVs in which the CPS of serotype 4 of the pneumococcus was chemically linked to recombinant carrier proteins of the pathogen. The pneumococcal proteins selected as carriers were NanA (neuraminidase), PiuA (iron uptake protein) and SP0148. The procedure involved cloning of recombinant genes expressing the capsule-encoding locus of serotype 4, together with genes encoding the test recombinant pneumococcal proteins, into *Escherichia coli* [95, 96]. Following induction of recombinant gene-expression, the experimental PCVs were generated using protein glycan coupling technology. Immunization of mice with all three of the purified experimental PCVs resulted in significant increases in the systemic levels of opsonophagocytic antibodies to both the CPS and protein carriers, with the PiuA conjugate showing equivalence to PCV13, with respect not only to antibody production, but also protection of mice against experimental infection.

In the second of these, Santiesteban-Lores and colleagues [97] described the development of experimental PCVs in which capsular oligosaccharides of molecular size 15–20 kDa (optimally immunogenic) prepared from weakly immunogenic serotype 6B were conjugated by a process of reductive amination to recombinant PspA clade 1, family 1 (PspA1) and PspA clade 3, family 2 (PspA3) proteins as a strategy to achieve maximum pneumococcal serotype coverage. Despite minor structural alterations to both the oligosaccharide and protein components of the vaccines, immunogenicity was not impaired by the conjugation process. Immunization of mice with both experimental PCVs resulted in significant production of IgG antibodies to both the CPS and PspA vaccine components. However, only antibodies to PspA possessed functional opsonophagocytic activity against the vaccine serotype, as well as against two unrelated serotypes of the pneumococcus, which also correlated with protection against experimental infection [97].

The third study, reported very recently by Guo et al. [98], described a protein/polysaccharide conjugation procedure, based on the multiple antigen-presenting system (MAPS) previously documented by Zhang et al., in which biotin/rhizavidin is utilized as a strategy to generate integrated, high-affinity, non-covalently bound macromolecular protein/polysaccharide complexes with vaccine potential [99]. In their study, Guo et al., non-covalently and efficiently, linked biotinylated CPS4 with the pneumococcal fusion proteins, PsaA – PspA2,3 and PspA4. In the case of PsaA – PspA2,3, this fusion protein combined the highly-conserved PsaA protein with PspA2,3 comprised of the αHD region of clade 2 PspA with the complementarity-determining region of clade 3 PspA, while PspA4 was comprised of the αHD and PRD regions of clade 4 PspA. Immunogenicity of the novel PCVs, PsaA-PspA2,3-CPS4 and PspA4-CPS4, was investigated using murine models of experimental pneumococcal infection. The authors observed the following: (i) immunization of mice with the experimental conjugates of CPS4 resulted in induction of levels of anti-CPS4 IgG antibodies, which were 17-fold and fivefold higher than those induced by purified, carrier-free CPS4 and PCV13, respectively; (ii) these IgG antibodies possessed functional opsonophagocytic activity, which induced complement-dependent killing of the pneumococcus that was more effective than that of antibodies induced by the individual CPS4 and protein components of the experimental PCVs, but less than that of antibodies induced by immunization with PCV13; (iii) the experimental vaccines induced Th1 and Th17 cell-mediated immune responses, which were characterized by increased production of tumor necrosis factor-α and interleukin-17A, respectively, by isolated, vaccine-activated splenocytes from pre-immunized mice; and (iv) mice immunized with the experimental PCVs exhibited a significantly increased strain-dependent survival rate that reached 90% following administration of a lethal dose of *S. pneumoniae* serotype 8, PspA family 1, clade 1, but only 35% following infection with serotype 4, PspA, family 2, clade 3, compared with a survival rate of 20% in the case of mice immunized with PCV 13.

However, the most advanced and versatile of these protein/polysaccharide hybrid vaccines is undoubtedly the 24-valent ASP3772 (to be renamed AFX3772 following reacquisition of the exclusive world rights by Affinivax Inc.) [100]. As the name implies, ASP3772 contains 24 different pneumococcal CPS, including those present in PCV13 and PPSV23 (with the exception of serotype 20, as well as serotype 6A in the case of PPSV23). Each CPS is biotinylated and merged with rhizavidin-bound protein fragments derived from the pneumococcal conserved surface proteins, SP0785 and SP1500 [100].

ASP3772 has been evaluated for safety and immunogenicity relative to PCV13 and PPSV23 in a phase I/II clinical trial, which encompassed healthy adults aged 18–64 years and older adults aged 65–85 years, the results of which have been published recently [100]. With respect to safety, ASP3772 was well tolerated, while immunogenicity was notable, with “robust” immune responses evident across all age groups being superior to all PPV23 serotypes, as well several PCV13 serotypes. The authors concluded that ASP3772 “is highly immunogenic, and in adults may offer significantly broader protection than existing pneumococcal vaccines” [100]. On the basis of the positive findings of this clinical trial, the US Food and Drug Administration awarded ASP3772 breakthrough therapy designation.

These studies [96–98, 100] clearly demonstrate the potential of recombinant pneumococcal proteins to serve as carriers of CPS in the manufacture of novel PCVs, which may confer increased immunogenicity and broader pneumococcal coverage than traditional PCVs. Although promising, particularly in the case of ASP3772, these are nevertheless, early, preclinical studies, which do not encompass either clinical efficacy, or the logistics and costs of vaccine production.

### Other types of protein-based pneumococcal vaccines under development/evaluation

These include delivery of genetic material encoding recombinant pneumococcal proteins using DNA vaccine strategies. To date, however, we are aware of only very limited studies of this type. These include an earlier study, which described an attenuated *Salmonella* DNA vaccine that delivered recombinant PspA, which, in turn, protected mice experimentally infected with influenza virus against secondary pneumococcal infection [101]. Future innovations include the possible development of messenger RNA (mRNA) vaccines [86].

There is also ongoing interest in the development of inactivated, non-encapsulated *S. pneumoniae* whole cell vaccines, specifically one that is based on strain RM200 RX1E PdT AlytA [102]. This strain of the pneumococcus has been genetically manipulated to generate a detoxified pneumolysin mutant in which the autolysin gene has also been deleted. This vaccine, which is adsorbed to aluminum hydroxide and administered intramuscularly, has successfully completed a phase I clinical trial. PnuBioVax is another type of genetically modified, pneumococcal whole cell vaccine in which the *ply* gene of *S. pneumoniae* (TIGR4) has been mutated with retention of immunogenicity, while a temperature shift causes a stress-mediated transition from a commensal to an invasive phenotype, which is associated with augmentation of the surface proteins, PspA, PiaA, PiuA RrgA and RrgB (both pilus proteins), as well as Ply [103]. This vaccine has demonstrated safety and immunogenicity in a phase I clinical trial [103]. Another whole cell vaccine, currently in the preclinical stages of development, is based on gamma irradiation of a mutated, non-encapsulated Rx1 strain of the pneumococcus [104].

Despite acceptable safety and immunogenicity data, as well as apparent potential to prevent nasopharyngeal colonization by the pneumococcus [105, 106], concerns remain about the ability of whole cell vaccines of this type to protect against fully encapsulated, invasive pneumococci, in which surface proteins are not exposed to the immune defenses of the immunized host. Similar concerns, as well as safety issues, also apply to recently described experimental vaccines based on the administration of bacterial lysates [106].

Although the predominantly protein pneumococcal experimental vaccines described in this section of the review (Table 7) have performed well in preclinical studies, with a number having advanced to early phase clinical evaluation, disappointingly none of the individual recombinant vaccines, or combinations of these, as well as the protein-boosted PVCs, has yet, to our knowledge, progressed to licensure. The disappointing efficacy of these various protein-based vaccines does not appear, however, to be attributable to poor immunogenicity, but rather to concealment of vaccine-targeted pneumococcal surface antigens by the thick, polysaccharide capsule of the pathogen, favoring nasopharyngeal colonization of the pathogen [89]. This mechanism may, however, be less effective during capsule shedding, an event which promotes translocation of the pathogen to the lower airways during invasive disease, although the efficacy of this process may depend on factors such as the serotype of the pathogen and the magnitude of shedding, as well as the immunogenicity of the exposed surface antigens. Irrespective of the mechanisms underpinning the limited protective efficacy of protein-based pneumococcal vaccines, ongoing effort and innovation may overcome the challenges confronting development of effective serotype-independent pneumococcal vaccines. In this context, the early promise shown by ASP3772 is reassuring.

**Table 7:** Types of putative recombinant pneumococcal protein-based vaccines

• Exclusively protein-based, containing detoxified recombinant pneumococcal proteins individually or in combination
• Fusion/hybrid vaccines alone or packaged in nanoparticles/nanogels
• PCVs boosted with added recombinant pneumococcal proteins
• Novel PCVs based on recombinant pneumococcal carrier proteins
• Inactivated, non-encapsulated whole cell vaccines manipulated to express native, as well as a limited number of recombinant proteins
• Early phase nucleic acid vaccines (DNA and mRNA) expressing recombinant pneumococcal proteins

## Conclusions

Following the discovery of the pneumococcus in 1881, research has focused on the microbiology, epidemiology, pathogenesis and, most importantly in the context of the current review, development of effective immunization strategies to protect against pneumococcal infection. The earliest vaccines were based on the inoculation of heat-killed, mixed serotypes of the pneumococcus. However, following recognition of distinct serotypes of the pathogen, as well as the primary immunogenicity of CPS in the induction of protective antibody-mediated immune responses, subsequent vaccine development was based on formulations comprised of serotype-specific, isolated CPS. Following the discovery of penicillin, however, further pneumococcal vaccine development stagnated for almost 25 years until it was revived and passionately driven by Robert Austrian, whose efforts resulted in the licensing in 1983 of a purified capsular polysaccharide vaccine based on the 23 most common, disease-causing serotypes of the pathogen, PPSV23. This initiative paved the way for development of a series of PCVs that were immunogenic in young children. These PCVs have been integrated into national childhood immunization programs worldwide. Nevertheless, despite the success of PCVs in reducing pneumococcal carriage and invasive disease, particularly in children, as well as in high-risk adults, acquisition of a broadly serotype-independent vaccine remains the “holy grail” of pneumococcal vaccine development, albeit a challenging and difficult endeavor.

## Data Availability

This is a literature review and all studies and data are available on the internet.

## References

[1] Peyrani P, Mandell L, Torres A, Tillotson GS (2019). The burden of community-acquired bacterial pneumonia in the era of antibiotic resistance. Expert Rev Respir Med.

[2] Ferreira-Coimbra J, Sarda C, Rello J (2020). Burden of community-acquired pneumonia and unmet clinical needs. Adv Ther.

[3] GBD 2016 Lower Respiratory Infections Collaborators (2018). Estimates of the global, regional, and national morbidity, mortality, and aetiologies of lower respiratory infections in 195 countries, 1990–2016: a systematic analysis for the Global Burden of Disease Study 2016. Lancet Infect Dis.

[4] Feldman C, Anderson R (2020). Recent advances in the epidemiology and prevention of *Streptococcus pneumoniae* infections. F100Res..

[5] Austrian R, Gold J (1964). Pneumococcal bacteremia with especial reference to bacteremic pneumococcal pneumonia. Ann Intern Med.

[6] Sandvall B, Rueda AM, Musher DM (2013). Long-term survival following pneumococcal pneumonia. Clin Infect Dis.

[7] DiNardo AR, Netea MG, Musher DM (2021). Postinfectious epigenetic immune modifications - a double-edged sword. N Engl J Med.

[8] Lister S. The use of pneumococcal vaccine. South African Med Record. 1924;22(6):115–22.

[9] Wright AE, Morgan WP, Colebrook L, Dodgson RW (1914). Observations on prophylactic inoculation against pneumococcus infections, and on the results which have been achieved by it. Lancet.

[10] Barach AL (1928). Factors involved in the production of immunity with pneumococcus vaccine. I. Active and passive immunity during the first seven days after injection of antigen. J Exp Med..

[11] Klemperer G, Klemperer F. Versuche uber immunisirung und heilung bei der pneumokokkeninfection. Berlin Klin Wochenschrift. 1891;28(35):833–5; 869–75.

[12] Gray BM, Musher DM, Siber GR, Klugman KP, Mäkelä PH (2008). The history of pneumococcal disease. Pneumococcal Vaccines: The Impact of Conjugate Vaccine.

[13] White  B (1938). The Biology of Pneumococcus. The Bactereriologic, Biochemical and Immunological Characters and Activities of *Diplococcus pneumoniae*.

[14] Dochez AR, Gillespie LJ (1913). A biologic classification of pneumococci by means of immunity reactions. JAMA.

[15] Maynard GD (1915). Pneumonia inoculation experiment No III. Med J South Africa.

[16] Lister S (1924). The use of pneumococcal vaccine. South African Med Record.

[17] Lister S (1924). The use of pneumococcal vaccine. South African Med Record.

[18] Cecil RL, Austin JH (1918). Results of prophylactic inoculation against pneumococcus in 12,519 Men. J Exp Med.

[19] Dochez AR, Avery OT (1917). Soluble substance of pneumococcus origin in the blood and urine during lobar pneumonia. Exp Biol Med.

[20] Dochez AR, Avery OT (1917). The elaboration of specific soluble substance by pneumococcus during growth. J Exp Med.

[21] Heidelberger M, Avery OT (1923). The soluble specific substance of pneumococcus. J Exp Med.

[22] Heidelberger M, Avery OT (1924). The soluble specific substance of pneumococcus: second paper. J Exp Med.

[23] Felton LD (1934). Studies on the immunising substances in pneumococci. J Immunol.

[24] Ekwurzel GM, Simmons JS, Dublin LI, Felton LD (1938). Studies on immunizing substances in pneumococci. VIII. Report on field tests to determine the value of a pneumococcus antigen. Public Health Rep..

[25] Smillie WG, Wornock GH, White HJ (1938). A study of a type I pneumococcus epidemic at the State Hospital at Worcester. Mass Am J Pub Health.

[26] MacLeod CM, Hodges RG, Heidelberger M, Bernhard WG (1945). Prevention of pneumococcal pneumonia by immunization with specific capsular polysaccharides. J Exp Med.

[27] Shoar S, Musher DM (2020). Etiology of community-acquired pneumonia in adults: a systematic review. Pneumonia (Nathan).

[28] Maxwell AR, Lecture F (1975). Random gleanings from a life with the pneumococcus. J Infect Dis.

[29] Austrian R, Douglas RM, Schiffman G, Coetzee AM, Koornhof HJ, Hayden-Smith S (1976). Prevention of pneumococcal pneumonia by vaccination. Trans Assoc Am Physicians.

[30] Smit P, Oberholzer D, Hayden-Smith S, Koornhof HJ, Hilleman MR (1977). Protective efficacy of pneumococcal polysaccharide vaccines. JAMA.

[31] Moberley SA, Holden J, Tatham DP, Andrews RM (2008). Vaccines for preventing pneumococcal infection in adults. Cochrane Database Syst Rev..

[32] Huss A, Scott P, Stuck AE, Trotter C, Egger M (2009). Efficacy of pneumococcal vaccination in adults: a meta-analysis. CMAJ.

[33] Moberley S, Holden J, Tatham DP, Andrews RM (2013). Vaccines for preventing pneumococcal infection in adults. Cochrane Database Syst Rev..

[34] Simberkoff MS, Cross AP, Al-Ibrahim M, Baltch AL, Geiseler PJ, Nadler J (1986). Efficacy of pneumococcal vaccine in high-risk patients. Results of a veterans administration cooperative study. N Engl J Med..

[35] Shapiro ED, Berg AT, Austrian R, Schroeder D, Parcells V, Margolis A (1991). The protective efficacy of polyvalent pneumococcal polysaccharide vaccine. N Engl J Med.

[36] Suzuki M, Dhoubhadel BG, Ishifuji T, Yasunami M, Yaegashi M, Asoh N (2017). Adult pneumonia study group-Japan (APSG-J). Serotype-specific effectiveness of 23-valent pneumococcal polysaccharide vaccine against pneumococcal pneumonia in adults aged 65 years or older: a multicentre, prospective, test-negative design study. Lancet Infect Dis..

[37] Djennad A, Ramsay ME, Pebody R, Fry NK, Sheppard C, Ladhani SN (2019). Effectiveness of 23-valent polysaccharide pneumococcal vaccine and changes in invasive pneumococcal disease incidence from 2000 to 2017 in those aged 65 and over in England and Wales. EClinicalMedicine.

[38] Kim JH, Chun BC, Song JY, Kim HY, Bae IG, Kim DM (2019). Direct effectiveness of pneumococcal polysaccharide vaccine against invasive pneumococcal disease and non-bacteremic pneumococcal pneumonia in elderly population in the era of pneumococcal conjugate vaccine: A case-control study. Vaccine.

[39] Lawrence H, Pick H, Baskaran V, Daniel P, Rodrigo C, Ashton D (2020). Effectiveness of the 23-valent pneumococcal polysaccharide vaccine against vaccine serotype pneumococcal pneumonia in adults: a case-control test-negative design study. PLoS Med.

[40] Vila-Corcoles A, Hospital I, Ochoa-Gondar O, Satue E, de Diego C, Vila-Rovira A (2020). Clinical effectiveness of 13-valent and 23-valent pneumococcal vaccination in middle-aged and older adults: The EPIVAC cohort study, 2015–2016. Vaccine.

[41] Masuda T, Nakatani E, Shirai T, Akamatsu T, Tamura K, Takahashi S (2021). Effectiveness of a 23-valent pneumococcal polysaccharide vaccine for the prevention of pneumococcal pneumonia in the elderly with chronic respiratory diseases: a case-control study of a single center. BMC Pulm Med.

[42] Schneerson R, Barrera O, Sutton A, Robbins JB (1980). Preparation, characterization, and immunogenicity of Haemophilus influenzae type b polysaccharide-protein conjugates. J Exp Med.

[43] Black S, Shinefield H, Fireman B, Lewis E, Ray P, Hansen JR (2000). Efficacy, safety and immunogenicity of heptavalent pneumococcal conjugate vaccine in children. Northern California Kaiser Permanente vaccine study center group. Pediatr Infect Dis J..

[44] Dagan R, Melamed R, Muallem M, Piglansky L, Greenberg D, Abramson O (1996). Reduction of nasopharyngeal carriage of pneumococci during the second year of life by a heptavalent conjugate pneumococcal vaccine. J Infect Dis.

[45] Lipsitch M (1997). Vaccination against colonizing bacteria with multiple serotypes. Proc Natl Acad Sci U S A.

[46] Pilishvili T, Lexau C, Farley MM, Hadler J, Harrison LH, Bennett NM (2010). Sustained reductions in invasive pneumococcal disease in the era of conjugate vaccine. J Infect Dis.

[47] Ahmed SS, Pondo T, Xing W, McGee L, Farley M, Schaffner W (2020). Early impact of 13-valent pneumococcal conjugate vaccine use on invasive pneumococcal disease among adults with and without underlying medical conditions-United States. Clin Infect Dis.

[48] Bonten MJ, Huijts SM, Bolkenbaas M, Webber C, Patterson S, Gault S (2015). Polysaccharide conjugate vaccine against pneumococcal pneumonia in adults. N Engl J Med.

[49] McLaughlin JM, Jiang Q, Isturiz RE, Sings HL, Swerdlow DL, Gessner BD (2018). Effectiveness of 13-valent pneumococcal conjugate vaccine against hospitalization for community-acquired pneumonia in older US adults: a test-negative design. Clin Infect Dis.

[50] Vadlamudi NK, Parhar K, Altre Malana KL, Kang A, Marra F (2019). Immunogenicity and safety of the 13-valent pneumococcal conjugate vaccine compared to 23-valent pneumococcal polysaccharide in immunocompetent adults: a systematic review and meta-analysis. Vaccine.

[51] Jackson LA, Gurtman A, van Cleeff M, Jansen KU, Jayawardene D, Devlin C (2013). Immunogenicity and safety of a 13-valent pneumococcal conjugate vaccine compared to a 23-valent pneumococcal polysaccharide vaccine in pneumococcal vaccine-naive adults. Vaccine.

[52] Smith KJ, Wateska AR, Nowalk MP, Raymund M, Nuorti JP, Zimmerman RK (2012). Cost-effectiveness of adult vaccination strategies using pneumococcal conjugate vaccine compared with pneumococcal polysaccharide vaccine. JAMA.

[53] Stoecker C, Kim L, Gierke R, Pilishvili T (2016). Incremental cost-effectiveness of 13-valent pneumococcal conjugate vaccine for adults age 50 years and older in the United States. J Gen Intern Med.

[54] Feldman C, Dlamini SK, Madhi SA, Meiring S, von Gottberg A, de Beer JC (2020). The cost-effectiveness of using pneumococcal conjugate vaccine (PCV13) versus pneumococcal polysaccharide vaccine (PPSV23), in South African adults. PLoS ONE.

[55] Nuorti JP, Whitney CG (2010). Updated recommendations for prevention on invasive pneumococcal disease among adults using the 23-valent pneumococcal polysaccharide vaccine (PPSV23). MMWR Recomm Rep..

[56] Pilishvili T, Bennett NM (2015). Pneumococcal disease prevention among adults: Strategies for the use of pneumococcal vaccines. Vaccine.

[57] Bennett NM, Whitney CG, Moore M, Pilishvili T, Dooling KL, CDC (2012). Use of 13-valent pneumococcal conjugate vaccine and 23-valent pneumococcal polysaccharide vaccine for adults with immunocompromising conditions: Recommendations of the Advisory Committee on Immunization Practices (ACIP). MMWR Morb Mortal Wkly Rep.

[58] Tomczyk S, Bennett NM, Stoecker C, Gierke R, Moore MR, Whitney CG (2014). Use of 13-valent pneumococcal conjugate vaccine and 23-valent pneumococcal polysaccharide vaccine among adults aged >65 years: recommendations of the advisory committee on Immunization practices (ACIP). MMWR Morb Mortal Wkly Rep.

[59] Kobayashi M, Bennett NM, Gierke R, Almendares O, Moore MR, Whitney CG (2015). Intervals between PCV13 and PPSV23 vaccines: Recommendations of the advisory committee on immunization practices (ACIP). MMWR Morb Mortal Wkly Rep.

[60] Matanock A, Lee G, Gierke R, Kobayashi M, Leidner A, Pilishvili T (2019). Use of 13-valent pneumococcal conjugate vaccine and 23-valent pneumococcal polysaccharide vaccine among adults aged ≥65 years: updated recommendations of the advisory committee on immunization practices. MMWR Morb Mortal Wkly Rep.

[61] Musher DM (2016). Should committees that write guidelines and recommendations publish dissenting opinions?. Mayo Clin Proc.

[62] Park SB, Kim HJ, Cheong HJ (2019). Environmental factors which can affect the burden of pneumococcal disease and the immune response to pneumococcal vaccines: the need for more precisely delineated vaccine recommendations. Expert Rev Vaccines.

[63] Izurieta P, Bahety P, Adegbola R, Clarke C, Hoet B (2018). Public health impact of pneumococcal conjugate vaccine infant immunization programs: assessment of invasive pneumococcal disease burden and serotype distribution. Expert Rev Vaccines.

[64] Papadatou I, Spoulou V (2016). Pneumococcal vaccination in high-risk individuals: are we doing it right?. Clin Vaccine Immunol.

[65] Garcia Garrido HM, Mak AMR, Wit F, Wong GWM, Knol MJ, Vollaard A (2020). Incidence and risk factors for invasive pneumococcal disease and community-acquired pneumonia in human immunodeficiency virus-infected individuals in a high-income setting. Clin Infect Dis.

[66] Nunes MC, von Gottberg A, de Gouveia L, Cohen C, Kuwanda L, Karstaedt AS (2011). Persistent high burden of invasive pneumococcal disease in South African HIV-infected adults in the era of an antiretroviral treatment program. PLoS ONE.

[67] Sadlier C, O'Dea S, Bennett K, Dunne J, Conlon N, Bergin C (2016). Immunological efficacy of pneumococcal vaccine strategies in HIV-infected adults: a randomized clinical trial. Sci Rep.

[68] Menéndez R, España PP, Pérez-Trallero E, Uranga A, Méndez R, Cilloniz C (2017). The burden of PCV13 serotypes in hospitalized pneumococcal pneumonia in Spain using a novel urinary antigen detection test. CAPA study Vaccine.

[69] Prato R, Fortunato F, Martinelli D (2016). Pneumococcal pneumonia prevention among adults: is the herd effect of pneumococcal conjugate vaccination in children as good a way as the active immunization of the elderly?. Curr Med Res Opin.

[70] van der Linden M, Perniciaro S, Imöhl M. 00091 Invasive pneumococcal disease among adults in Germany: the limit of herd protection. 29^th^ ECCMID 13–16 April 2019 Amsterdam, Netherlands. https://www.escmid.org/escmid_publications/escmid_elibrary/material/?mid=66609

[71] van der Linden M, Imöhl M, Perniciaro S (2019). Limited indirect effects of an infant pneumococcal vaccination program in an aging population. PLoS ONE.

[72] Torres A, Menéndez R, España PP, Fernández-Villar JA, Marimón JM, Cilloniz C (2021). CAPA Study Group. The evolution and distribution of pneumococcal serotypes in adults hospitalized with community-acquired pneumonia in Spain using a serotype-specific urinary antigen detection test: The CAPA Study, 2011–2018. Clin Infect Dis..

[73] GERMS-SA Annual Surveillance Review. 2019;8–49. Available from: https://www.nicd.ac.za/wp-content/uploads/2021/02/GERMS-Annual-Review-2019_.pdf

[74] Pelton SI, Bornheimer R, Doroff R, Shea KM, Sato R, Weycker D (2019). Decline in pneumococcal disease attenuated in older adults and those with comorbidities following universal childhood PCV13 immunization. Clin Infect Dis.

[75] Pelton SI, Shea KM, Farkouh RA, Strutton DR, Braun S, Jacob C (2015). Rates of pneumonia among children and adults with chronic medical conditions in Germany. BMC Infect Dis.

[76] Kobayashi M, Farrar JL, Gierke R, Britton A, Childs L, Leidner AJ (2022). Use of 15-valent pneumococcal conjugate vaccine and 20-valent pneumococcal conjugate vaccine among U.S. adults: Updated Recommendations of the Advisory Committee on Immunization Practices - United States, 2022. MMWR Morb Mortal Wkly Rep..

[77] Weinberger DM, Shapiro ED (2020). Pneumococcal vaccines for adults: what's next?. Clin Infect Dis.

[78] Klugman KP, Rodgers GL (2021). Time for a third-generation pneumococcal conjugate vaccine. Lancet Infect Dis.

[79] Rodgers GL, Whitney CG, Klugman KP (2021). Triumph of pneumococcal conjugate vaccines: overcoming a common foe. J Infect Dis.

[80] Principi N, Esposito S (2021). Pneumococcal disease prevention: are we on the right track?. Vaccines.

[81] Fairman J, Agarwal P, Barbanel S, Behrens C, Berges A, Burky J (2021). Non-clinical immunological comparison of a Next-Generation 24-valent pneumococcal conjugate vaccine (VAX-24) using site-specific carrier protein conjugation to the current standard of care (PCV13 and PPV23). Vaccine.

[82] McGuinness D, Kaufhold RM, McHugh PM, Winters MA, Smith WJ, Giovarelli C (2021). Immunogenicity of PCV24, an expanded pneumococcal conjugate vaccine, in adult monkeys and protection in mice. Vaccine.

[83] Feldman C, Anderson R (2014). Review: current and new generation pneumococcal vaccines. J Infect.

[84] Masomian M, Ahmad Z, Gew LT, Poh CL (2020). Development of next generation *Streptococcus pneumoniae* vaccines conferring broad protection. Vaccines.

[85] Scott NR, Mann B, Tuomanen EI, Orihuela CJ (2021). Multi-valent protein hybrid pneumococcal vaccines: a strategy for the next generation of vaccines. Vaccines.

[86] Oliveira GS, Oliveira MLS, Miyaji EN, Rodrigues TC (2021). Pneumococcal vaccines: past findings, present work, and future strategies. Vaccines.

[87] McDaniel LS, Swiatlo E (2021). If Not Now, When? Nonserotype Pneumococcal Protein Vaccines. Open Forum Infect Dis..

[88] Bologa M, Kamtchoua T, Hopfer R, Sheng X, Hicks B, Bixler G (2012). Safety and immunogenicity of pneumococcal protein vaccine candidates: monovalent choline-binding protein A (PcpA) vaccine and bivalent PcpA-pneumococcal histidine triad protein D vaccine. Vaccine.

[89] Zangari T, Zafar MA, Lees JA, Abruzzo AR, Bee GCW, Weiser JN (2021). Pneumococcal capsule blocks protection by immunization with conserved surface proteins. NPJ Vaccines.

[90] Wang Y, Xia L, Wang G, Lu H, Wang H, Luo S (2022). Subcutaneous immunization with the fusion protein ΔA146Ply-SP0148 confers protection against *Streptococcus pneumoniae* infection. Microb Pathog.

[91] Yuki Y, Uchida Y, Sawada S-I, Nakahashi-Ouchida R, Sugiura K, Mori H (2021). Characterization and specification of a trivalent protein-based pneumococcal vaccine formulation using an adjuvant-free nanogel nasal delivery system. Mol Pharm.

[92] Nakahashi-Ouchida R, Uchida Y, Yuki Y, Katakai Y, Yamanoue T, Ogawa H (2021). A nanogel-based trivalent PspA nasal vaccine protects macaques from intratracheal challenge with pneumococci. Vaccine.

[93] Odutola A, Ota MOC, Antonio M, Ogundare EO, Saidu Y, Foster-Nyarko E (2017). Efficacy of a novel, protein-based pneumococcal vaccine against nasopharyngeal carriage of *Streptococcus pneumoniae* in infants: A phase 2, randomized, controlled, observer-blind study. Vaccine.

[94] Hammitt LL, Campbell JC, Borys D, Weatherholtz RC, Reid R, Goklish N (2019). Efficacy, safety and immunogenicity of a pneumococcal protein-based vaccine co-administered with 13-valent pneumococcal conjugate vaccine against acute otitis media in young children: A phase IIb randomized study. Vaccine.

[95] Reglinski M, Ercoli G, Plumptre C, Kay E, Petersen FC, Paton JC (2018). A recombinant conjugated pneumococcal vaccine that protects against murine infections with a similar efficacy to Prevnar-13. NPJ Vaccines.

[96] Kay EJ, Yates LE, Terra VS, Cuccui J, Wren BW (2016). Recombinant expression of *Streptococcus pneumoniae* capsular polysaccharides in *Escherichia coli*. Open Biol.

[97] Santiesteban-Lores LE, Cabrera-Crespo J, Carvalho E (2021). Development of a pneumococcal conjugate vaccine based on chemical conjugation of polysaccharide serotype 6B to PspA. Microb Pathog.

[98] Guo M, Guo X, Zhang C, Zhu S, Zhang Y, Gu T (2022). Novel pneumococcal protein-polysaccharide conjugate vaccine based on biotin-streptavidin. Infect Immun.

[99] Zhang F, Lu YJ, Malley R (2013). Multiple antigen-presenting system (MAPS) to induce comprehensive B- and T-cell immunity. Proc Natl Acad Sci U S A.

[100] Chichili GR, Smulders R, Santos V, Cywin B, Kovanda L, Van Sant C (2022). Phase 1/2 study of a novel 24-valent pneumococcal vaccine in healthy adults aged 18 to 64 years and in older adults aged 65 to 85 years. Vaccine.

[101] Seo SU, Kim JJ, Yang H, Kwon HJ, Yang JY, Curtiss Iii R (2012). Effective protection against secondary pneumococcal pneumonia by oral vaccination with attenuated Salmonella delivering PspA antigen in mice. Vaccine.

[102] Keech CA, Morrison R, Anderson P, Tate A, Flores J, Goldblatt D (2020). A phase 1 randomized, placebo-controlled, observer-blinded trial to evaluate the safety and immunogenicity of inactivated *Streptococcus pneumoniae* whole-cell vaccine in adults. Pediatr Infect Dis J.

[103] Entwisle C, Hill S, Pang Y, Joachim M, McIlgorm A, Colaco C (2017). Safety and immunogenicity of a novel multiple antigen pneumococcal vaccine in adults: a Phase 1 randomised clinical trial. Vaccine.

[104] David SC, Laan Z, Minhas V, Chen AY, Davies J, Hirst TR (2019). Enhanced safety and immunogenicity of a pneumococcal surface antigen a mutant whole-cell inactivated pneumococcal vaccine. Immunol Cell Biol.

[105] Roy EM, Zhang F, Malley R, Lu YJ (2022). Induction of T cell responses by vaccination of a *Streptococcus pneumoniae* whole-cell vaccine. Methods Mol Biol.

[106] Ferrara F, Rial A, Suárez N, Chabalgoity JA (2021). Polyvalent bacterial lysate protects against pneumonia independently of neutrophils, IL-17A or Caspase-1 activation. Front Immunol.

